# Amorphous Silicon Nanowires Grown on Silicon Oxide Film by Annealing

**DOI:** 10.1186/s11671-017-2251-1

**Published:** 2017-08-10

**Authors:** Zhishan Yuan, Chengyong Wang, Ke Chen, Zhonghua Ni, Yunfei Chen

**Affiliations:** 1 School of Electromechanical Engineering, Guangdong University of Technology, Guangzhou, 510006 China; 2 Jiangsu Key Laboratory for Design and Manufacture of Micro-Nano Biomedical Instruments, Southeast University, Nanjing, 211189 China

**Keywords:** α-SiNWs, Cu patterns, Annealing time, Resistivity

## Abstract

In this paper, amorphous silicon nanowires (α-SiNWs) were synthesized on (100) Si substrate with silicon oxide film by Cu catalyst-driven solid-liquid-solid mechanism (SLS) during annealing process (1080 °C for 30 min under Ar/H_2_ atmosphere). Micro size Cu pattern fabrication decided whether α-SiNWs can grow or not. Meanwhile, those micro size Cu patterns also controlled the position and density of wires. During the annealing process, Cu pattern reacted with SiO_2_ to form Cu silicide. More important, a diffusion channel was opened for Si atoms to synthesis α-SiNWs. What is more, the size of α-SiNWs was simply controlled by the annealing time. The length of wire was increased with annealing time. However, the diameter showed the opposite tendency. The room temperature resistivity of the nanowire was about 2.1 × 10^3^ Ω·cm (84 nm diameter and 21 μm length). This simple fabrication method makes application of α-SiNWs become possible.

## Background

Among the various classes of one-dimensional semiconductor nanostructure, silicon nanowire (SiNW) has been exhibited bright future in the fields of electronic, photovoltaic solar, photonic, battery, and sensor. [1–6] The SiNW manufacture method includes top-down and bottom-up approaches. Table 1 is the summary of different SiNW manufacture method. Top-down approach is usually realized by reactive ion etching (RIE) and metal-catalyzed electroless etching of silicon. In those methods, nanowire site is controlled in top-down approach by nanofabrication tools such as e-beam lithography, [7] nanoimprint lithography [8], or nanosize template such as PS sphere, [9] AAO mask [10]. Nanofabrication tools control the site, size, orientation, and numbers of wire well with high-cost and complex fabrication process. Nanosize template [9–11] is the low-cost method, but the fabrication process is more complex than nanofabrication tool method for template should be built and removed during the whole process. Therefore, template-free method shows good potential in future [12]. Another top-down approach uses MEMS technique to fabricate site controllable SiNWs [13], this fabrication process easily fabricate SiNW sensor devices. However, MEMS technique brings complex manufacture process with high cost.

**Table 1:** Summary of different SiNW manufacture method

Manufacture method		Advantages	Disadvantages	References
Top-down	RIE	The site and size of nanowires were well controlled.	Need nanofabrication tools.	[7–10]
	Metal-catalyzed electroless etching		Template fabrication process was complex.	[11]
	MEMS technique	The site and size of nanowires were well controlled, without any nanofabrication tools.	Complex fabrication process and time-consuming.	[13]
Bottom-up	CVD	Simple and low cost and the quality of SiNW was good.	Novel metal materials were prohibited in clean rooms. Controllability is poor.	[14–16]
	OAG	Simple and low cost and no metal catalyst is needed.	Controllability is poor. The compatibility with Si-based integration technology was poor.	[17, 18]
	Laser ablation			[19]
	SiO evaporation			[20]

In bottom-up approach, chemical vapor deposition (CVD) is an important approach to synthesis SiNWs with low-cost and simple fabrication process. And this approach can readily produce extremely small diameter and super long SiNWs (as recorded, the smallest diameter was 1 nm, and the longest was millimeters) [14–16]. Good quality SiNWs are always synthesized through vapor-liquid-solid (VLS) mechanism with the help of Au or other metals in this method [2]. However, those novel materials are prohibited in clean rooms for degrading the electrical and optical properties of semiconductors.

Catalyst free method is put forward to solve pollution problem which brought by novel catalysts in bottom-up approach. Oxide-assisted growth (OAG) method does not require any metal catalyst [17]. Unfortunately, the compatibility with Si-based integration technology is poor in this method. And products are always affected by other residual impurities easily [18]. Room temperature continuous wave laser ablation of Si is another way to synthesis SiNWs without using metal catalyst [19]. Nevertheless, high vacuum is needed. Even in the simple SiO evaporation technique, good size controllability is always hard to realize. Moreover, SiO powder is harmful to health [20].

New catalysts such as aluminum and copper are researched to open the door of complementary metal oxide semiconductor (CMOS) technology to SiNWs [21]. Aluminum is used to reduce the deep level impurities; it can also be a p-type dopant producing a shallow acceptor in Si. However, the high sensitivity to oxidation makes using aluminum as catalyst method becoming unpractical. Copper is a good conductor of heat and electricity and has been widely used in integrated circuits (ICs) and CMOS processing. So, copper is considered as the suitable catalyst for SiNW growth. The size and site of Si wires were well controlled by copper catalyst in Kayes et al. work [22]. In the works which copper was used as catalyst to synthesis SiNWs, SiH_4_, Si_2_H_6_, or SiCl_4_ gases were used as Si precursor [22–24].

In this paper, we present a simple and effective method to synthesis SiNWs on SiO_2_ films by Cu catalyst-driven SLS mechanism during annealing process without using any toxic precursor gases. This method has two advantages. Firstly, the metal contamination of the SiNWs was decreased. Secondly, no toxic precursor gases were used.

## Methods

### Chip Fabrication

**Fig. 1:**
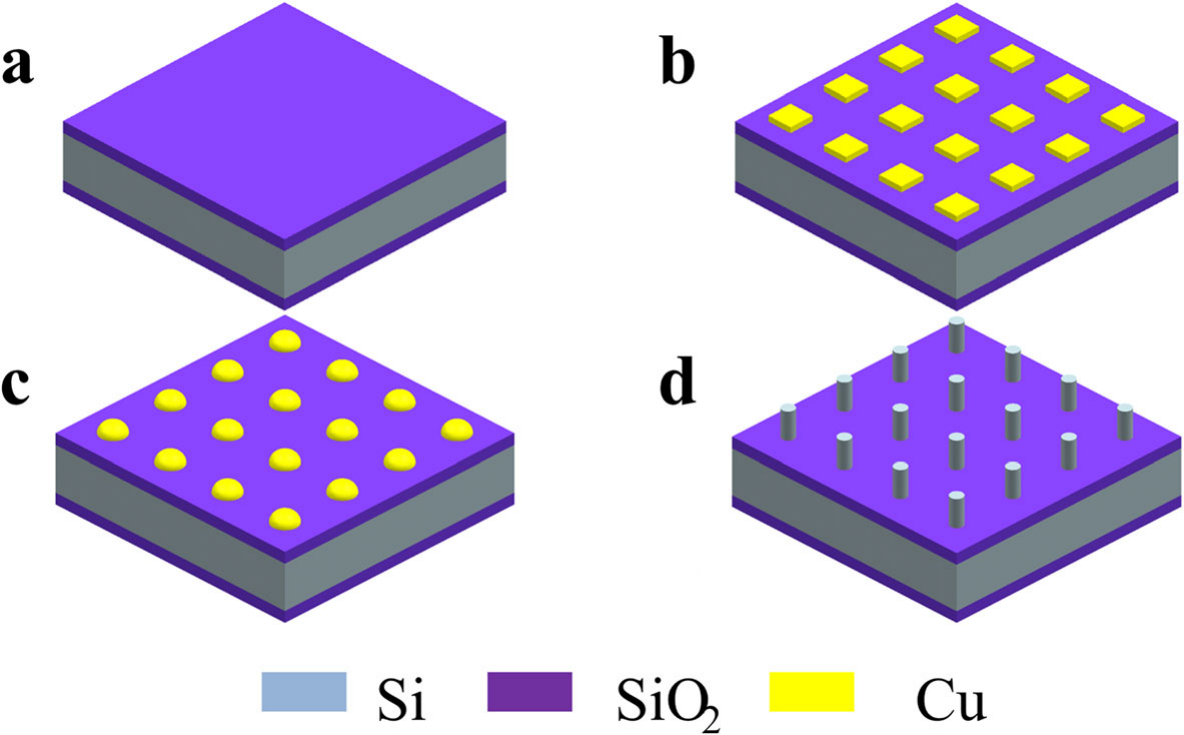
Schematic depiction of the fabrication process. **a** Thermal oxidation. **b** Cu micron-size pattern array fabrication. **c** Copper patterns changed into hemispheres. **d** Nanowire growth

First, 300 nm SiO_2_ film was grown on single side polished n-type silicon (100) wafers by thermal oxidation (Fig. 1a). Then, 400 nm copper film was deposited on SiO_2_ by magnetron sputtering. After photolithographic process and ammonium persulfate solution (1:100 water) etching, Cu micron-size pattern array were fabricated on SiO_2_ surface in target area (Fig. 1b). Subsequently, the wafer was diced into chips. And those chips were ultrasonically cleaned by ethanol and acetone in turn for 10 min. Afterwards, DI water was used for last clean process before blow-dry by N_2_.

### α-SiNW Growth

One thousand standard cubic centimeters per minute of Ar was used to exclude air in the tube for 10 min after chips were put on quartz boat and transferred into the center of the horizontal furnace.

**Fig. 2:**
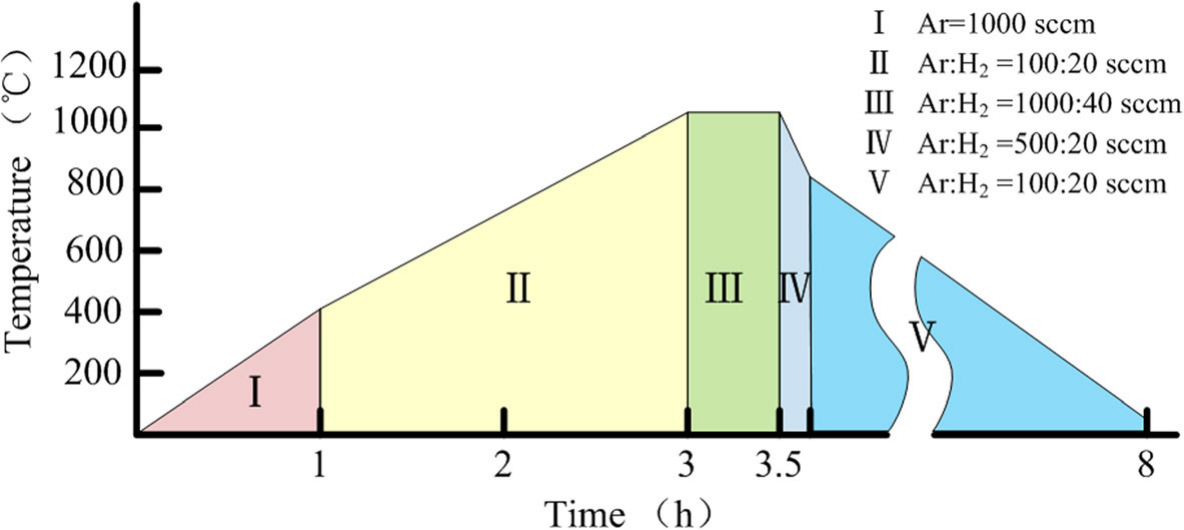
Thermal processing conditions for SiNW synthesis using a horizontal furnace. In stage I, the temperature was increased from room temperature to 400 °C in 1 h with the same Ar flow which used to exclude air. In stage II, Ar flow was adjusted to 100 sccm, and 20 sccm H_2_ was added. It took 2 h to reach 1080 °C. In this stage, copper patterns changed into hemispheres. Then, temperature was held for 30 min with 1000 sccm Ar and 40 sccm H_2_ in stage III. After turning off the furnace, the fast cooling process only 10 min was taken as the IV stage and the flow was adjusted to 500 and 20 sccm respectively. In the last stage, slow cooling used to decrease the furnace temperature to room temperature with 100 sccm Ar and 20 sccm H_2_

Subsequently, five stages were used to synthesis SiNWs. The detailed annealing processing conditions are given in Fig. 2. In stage I, the temperature was increased from room temperature to 400 °C in 1 h with the same Ar flow which is used to exclude air. In stage II, Ar flow was adjusted to 100 sccm, and 20 sccm H_2_ was added. It took 2 h to reach 1080 °C. In this stage, copper patterns changed into hemispheres (Fig. 1c). Then, temperature was held for 30 min with 1000 sccm Ar and 40 sccm H_2_ in stage III. After turning off the furnace, the fast cooling process of only 10 min was taken as the IV stage and the flow was adjusted to 500 and 20 sccm respectively. In the last stage, slow cooling used to decrease the furnace temperature to room temperature with 100 sccm Ar and 20 sccm H_2_. After the five stages, α-SiNWs were grown on the position of Cu patterns as shown in Fig. 1d.

### Characterization

Scanning electron microscopy (SEM, Hitachi S-4800) and high-resolution transmission electron microscopy (TEM, JEM-2100F operating at 200 Kv) equipped with energy dispersive spectrometer (EDS) were employed for analyzing the morphology and composition of the nanowires. For TEM measurements, Mo grid was used to support nanowires. For FIB etching the root of the wire, a thin layer of Au was evaporated on the surface to protect the wire by electron-beam-induced deposition (EBID). Two-terminal device was used to measurement the resistivity of nanowire [25]. The wire was mechanically removed from the substrate by nano-operator equipped on focused ion beam (FIB) (FEI, QUANTA3D 600FIB System). Then, nanowire was weld on the two electrodes by Pt deposited with assisted electron beam. Finally, the resistivity of the nanowire was measured by Cascade Semi-automatic probe station HP 4156.

## Results and Discussion

**Fig. 3:**
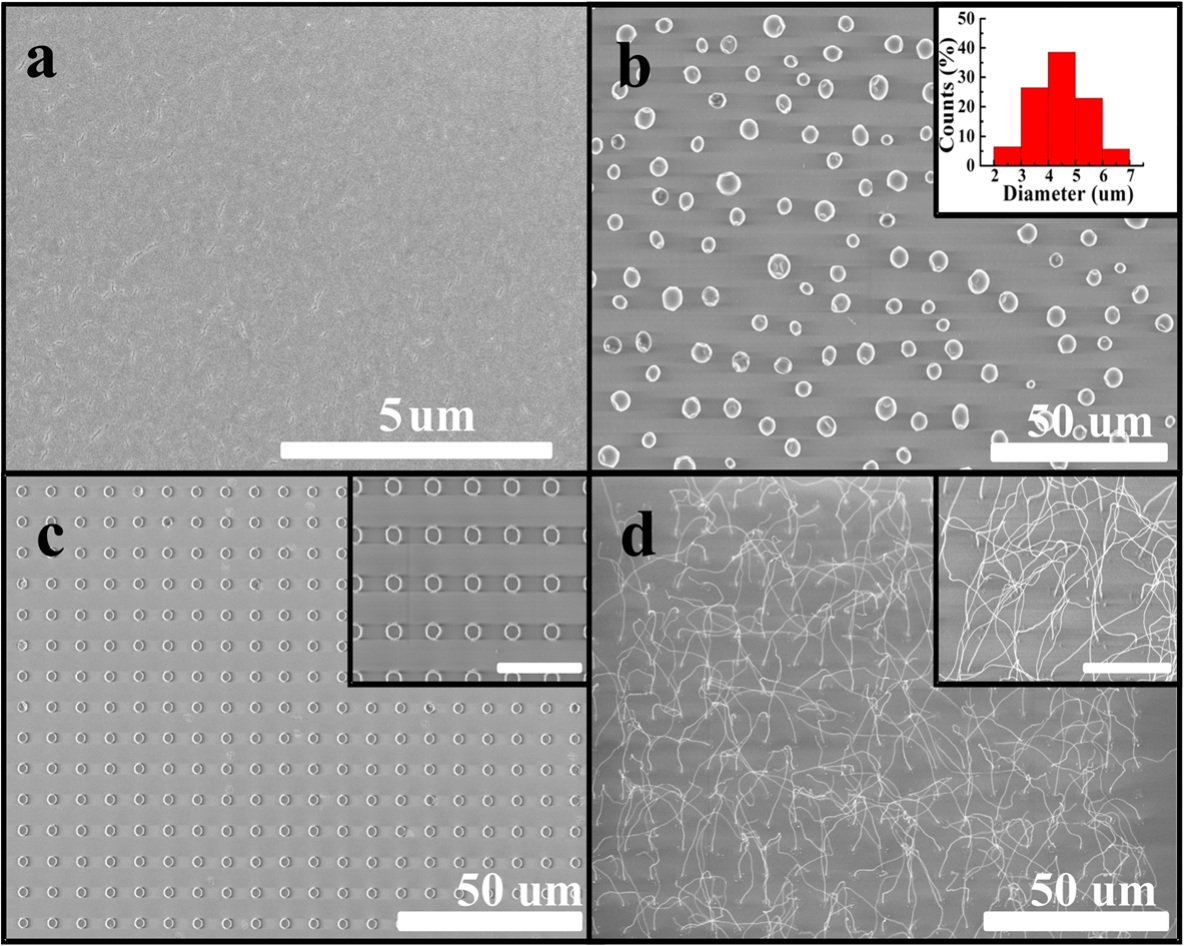
SEM images for the two samples on 300 nm SiO_2_ surface before and after 30 min annealed in Ar/H_2_ atmosphere at temperature of 1080 °C. **a** Pre-annealed SEM image of sample I with Cu nanofilm (400 nm thick). **b** SEM images of sample I with Cu film after annealed. *Inset photo* was the diameter distribution of the Cu particles after SEM. **c** Pre-annealed SEM image of sample II with Cu patterns array (Cu pattern size, 400-nm thick and 1.9 μm diameter). *Inset photo* was the magnified image of Cu patterns array. **d** SEM image of nanowire growth on sample II after annealed. *Inset photo* was the magnified image of nanowires. The *scale bars* in the *insets* are 10 μm

Figure 3 presents the SEM photos of two samples before and after annealing (sample I, the thick Cu film is 400 nm, sample II is the Cu pattern arrays with size of 400 nm thick and 1.9 μm diameter, and center-to-center pitch is 10 μm). It is obvious that the results of the two samples were quite different after annealed at 1080 °C for 30 min. For Cu film, shown in Fig. 3b, only Cu balls were scattered randomly on the surface of SiO_2_. The inserted figure in Fig. 3b was the diameter distributions of Cu balls, and the average diameter of the ball was 4.4 μm. In-suit nanowire appeared in sample II after annealing in Fig. 3d. The length of nanowire can be as long as 20 μm, and the diameter of nanowire is about 57 nm as shown in the inserted image of Fig. 3d. It is clear that each pattern has grown one nanowire and the center-to-center distance equal to the value of Cu patterns. This means the density of nanowires can be controlled by number of Cu patterns simply. The phenomenon in Fig. 3 demonstrates that the micro size of Cu patterns are suitable for nanowire growth (in our case, the size of Cu pattern was 400-nm thick and 1.9 μm diameter). For Cu film, dewetting effect happened at high temperature. In order to reduce the surface energy of Cu film, Cu balls were aggregated in random way (in Fig. 3b).

**Fig. 4:**
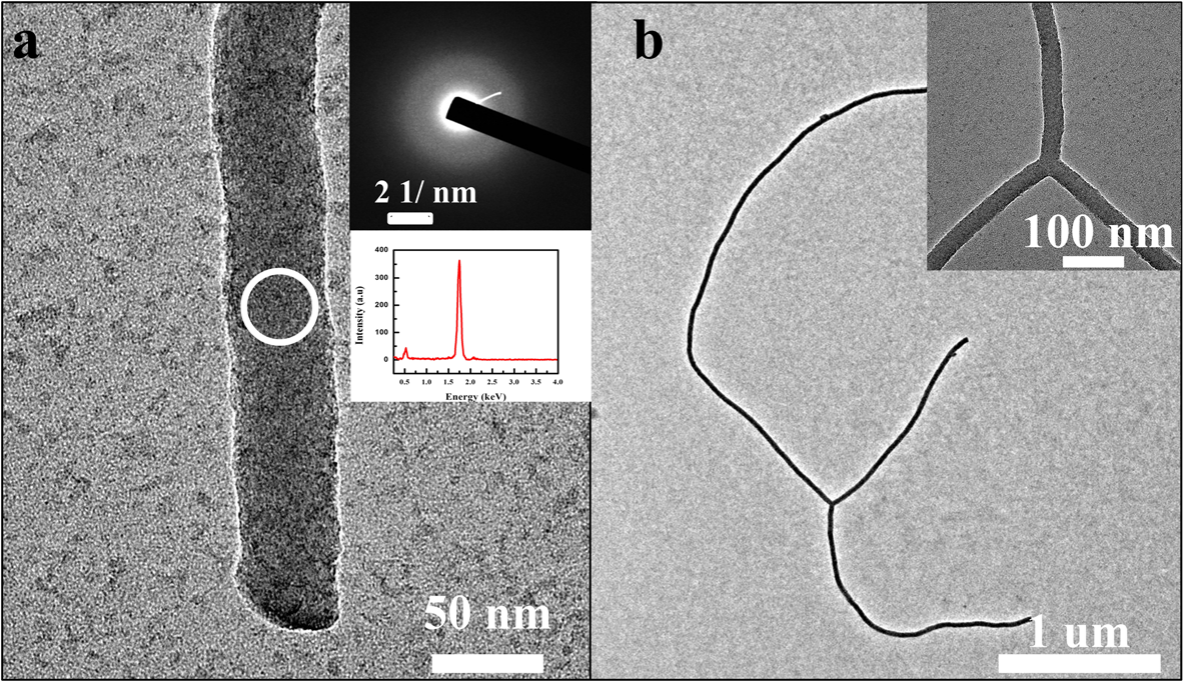
TEM images of nanowires. **a** TEM image of the tip part of nanowire. *Inset photos* were the selected area electron diffraction (SAED) of nanowire and the EDS spectrum respectively. The SAED pattern was obtained from the middle of the wire (*white circle*) in Fig. 4, and the aperture for SAED was 200 nm. **b** TEM image of nanowire. The inserted image was the detailed photo of nanowire in Fig. 4b

The high-resolution transmission electron microscopy (TEM) image in Fig. 4a reveals that the nanowire has a smooth morphology at diameter of 50 nm in sample II. The highly diffusive ring pattern (inset) of the selected area electron diffraction (SAED) demonstrates that the nanowire was totally amorphous (in Fig. 4). Energy dispersive spectrometer (EDS) results in Fig. 4 indicate that the wire consists of Si and O with atomic ratio of 4, which is far from the ratio of Si dioxide and suggests that a trace amount of oxygen exists in the SiNWs. For reduction atmosphere which was composed of Ar and H_2_ is maintained during nanowires growth process, so the light oxidiation only happened during sample exposure to air after fabrication.

**Fig. 5:**
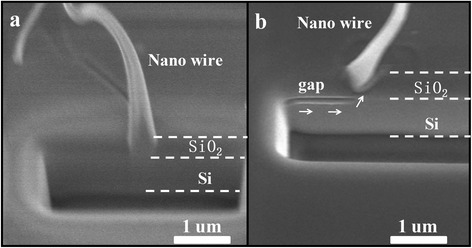
SEM images of α-SiNW root. **a**, **b** The SEM images of α-SiNW root part at tilt 45° after FIB etching. A long Si gap is found at the Si /SiO_2_ interface in (**b**)

**Fig. 6:**
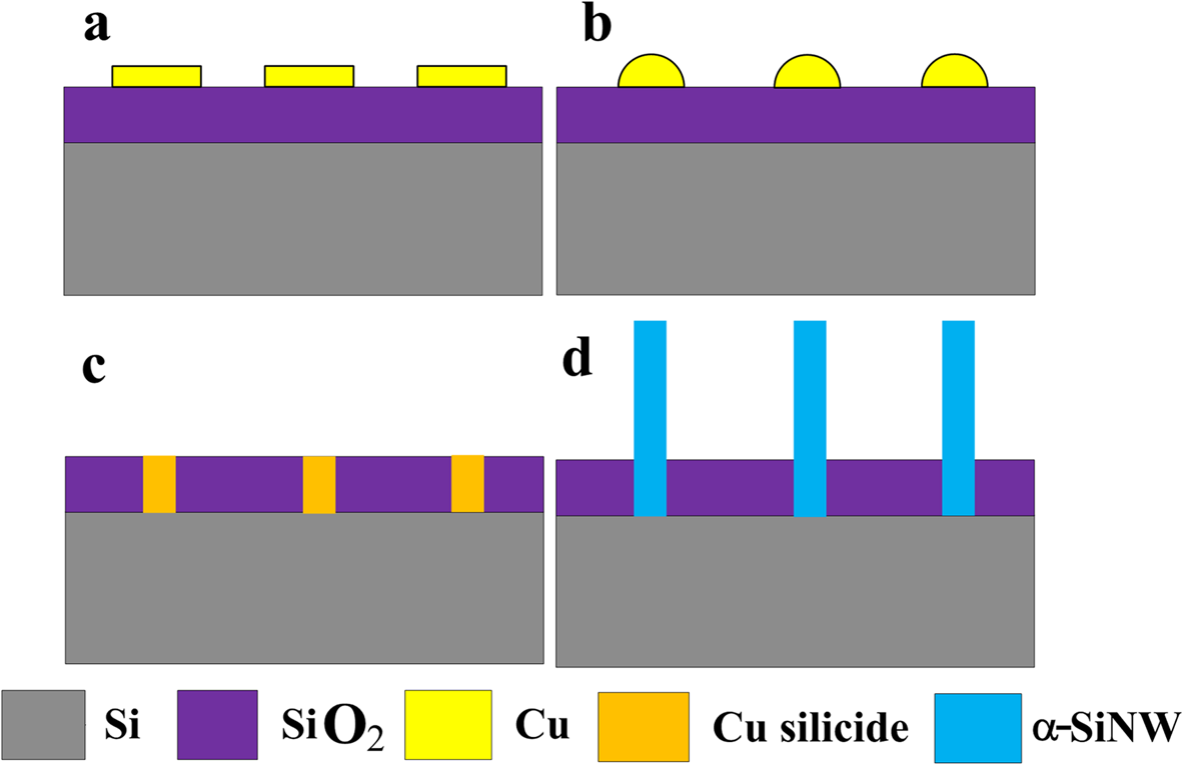
Schematic illustration of α-SiNW growth. During the annealing process, Cu patterns (**a**) dewet to the center of the pattern (**b**), and react with SiO_2_ to form Cu silicide (**c**). Then, Si atoms permeate into the Cu silicide. During this process, the different diffusion speed of Si atoms in the substrate which caused by the defect of substrate may induce the Si gap formation. When the dissolving Si atoms in silicide reached saturation, Si starts to precipitate to synthesis α-SiNWs (**d**)

After FIB etching the root part of the wire and substrate, cross section of the wire root was characterized by SEM with sample holder rotated 45°. It is interesting to find that the nanowire grown from the boundary between Si and SiO_2_ in Fig. 5. A long Si gap is also found at the Si /SiO_2_ interface. Those observations demonstrate that the substrate was the only Si source for the wire. Meanwhile, no metal particle is found at the tip part of the wire. According to those results, a possible schematic illustration of α-SiNW growth is presented in Fig. 6 based on solid-liquid-solid mechanism. During the annealing process, Cu patterns (Fig. 6a) dewet to the center of the pattern (Fig. 6b) and react with SiO_2_ to form Cu silicide (Fig. 6c). Then, Si atoms permeate into the Cu silicide. During this process, the different diffusion speed of Si atoms in the substrate which caused by the defect of substrate may induce the Si gap formation. When the dissolving Si atoms in silicide reached saturation, Si starts to precipitate to synthesis α-SiNWs (Fig. 6d).

**Fig. 7:**
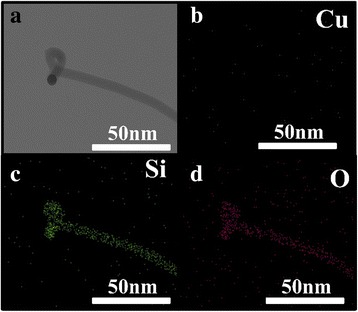
TEM and EDS mapping images of the tip part of the nanowire. **a** shows the TEM image of the tip part of the wire which seems like metal particle, **b**–**d** location of the different elements illustrated by EDS mapping with bright contrast variation: copper (**b**), silicon (**c**), and oxygen (**d**)

It is clear that Cu has played a very important role in our study. Something like black particle can be found at the tip of the wire, although in most wires, this particle is not existed. The mapping results (Fig. 7) show that no metal particle exists at the tip of the wire. The particle seems like the misunderstanding by the angle of between the wire and holder, which was not suitable to observe. Unfortunately, no copper can be found at the root part of the wire (Fig. 5). Cu diffused into Si substrates is the possible way that may give rise to this surprising result. It is well known that fast diffusion of Cu atoms in Si was tested at high temperature [26]. So, Cu atoms could diffuse into Si substrate in a few minutes after the window in SiO_2_ was opened at high temperature.

**Fig. 8:**
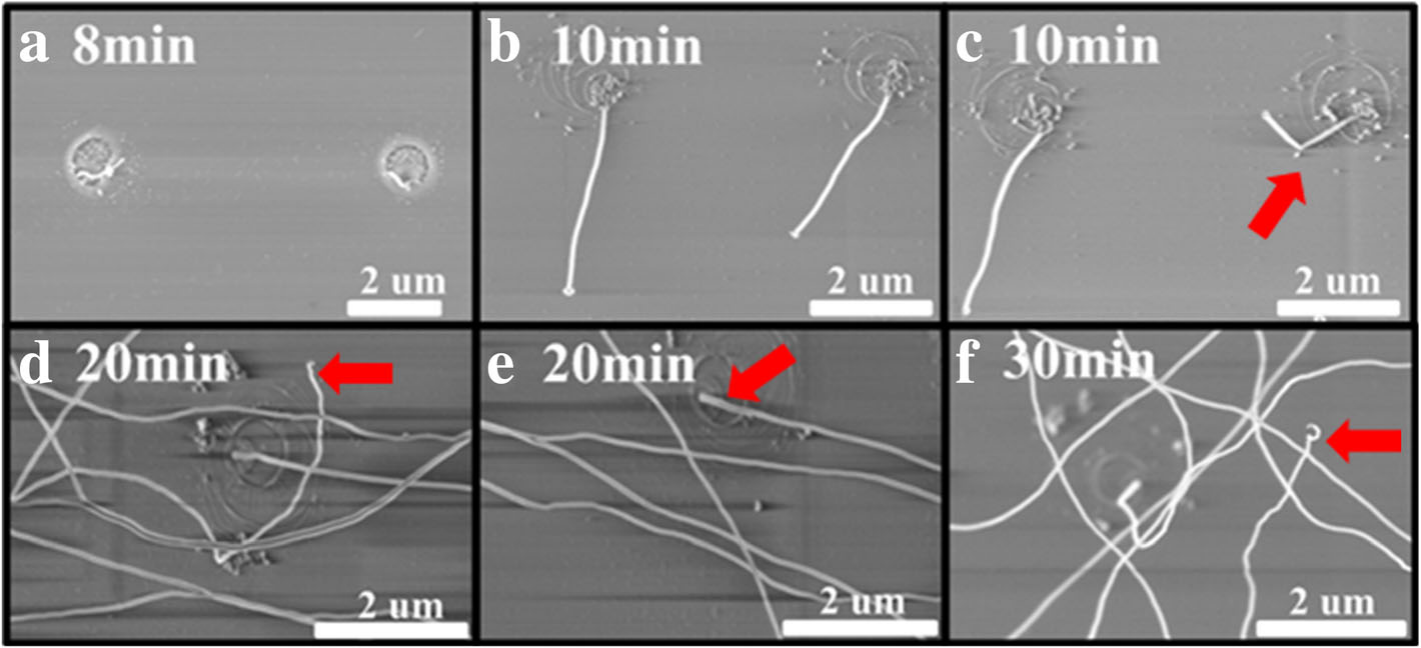
**a**–**f** The SEM images of α-SiNWs grown in different annealing time

To demonstrate the controllability of our method, different annealing time is carried out in our experiments. The SEM of nanowires synthesized in different annealing time with the same Cu pattern size (400-nm thick and 1.9 um diameter) and same SiO_2_ thickness (300 nm) is shown in Fig. 8. Most nanowires have uniform diameter. It is interesting to find that the diameter decreased when the direction of the wire changed. As red arrow shows in Fig. 8c, the tip part diameter is 76 nm, and the root part is only 49 nm. This huge difference in diameter in the same wire may be caused by the variation of the energy per unit area for the nucleus [27]. And this phenomenon is seldom to see. Another interest finding is that the diameter of root part was the bigger part in the whole wire, and the tip part was smaller (red arrows shown in d–f). Comparing with the whole wire, the length of nonuniform part is very short. This result presents that a-SiNW had nuniform diameter.

**Fig. 9:**
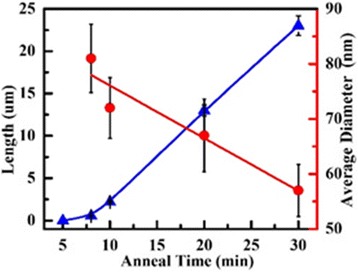
The nanowire diameter and length as a function of the anneal time. *Blue triangles* and *red circles* in figure are the date for length and diameter of nanowire in experiment, and *blue* and *red lines* in figure were the fitting line according to the experiment dates

After SEM, the length and diameter of root part of α-SiNWs are calculated. The results in Fig. 9 show that the length of α-SiNW was increased with annealing time, as a function of the anneal time. The diffusion time of Si atoms is increased offering more atoms to synthesis nanowire. The length of α-SiNW increases to 24 μm while the annealing time increased to 30 min. The average growth rate of nanowire is approximately 1.1 μm/min, which was similar to the growth rate by annealing with block Si source [28]. The rapid speed of growth is leaving no time for Si atoms to stack themselves into crystalline order. Finally, amorphous nanowires instead of crystalline are synthesized.

The diameter of α-SiNW is decreased from 81 to 57 nm in annealing time increasing process. Usually, the length of SiNW depends on their diameter for Gibbs-Thomson effect in vapor-liquid-solid growth using silane as gaseous source and gold as catalyst. The length of SiNW increases when diameter increased for nanowires with diameter under 100 nm. Nevertheless, the result in our experiment shows the inverse conclusion that the diameter decreased with length. Long-time annealing gives more time for Cu atoms diffused into Si substrate, and the volume of silicide catalyst is also decreased. Meanwhile, the diffusion process of Si atoms is continued which made growth of α-SiNW all the time with catalyst particle size change. Therefore, the diameter of α-SiNW is decreased with anneal time.

**Fig. 10:**
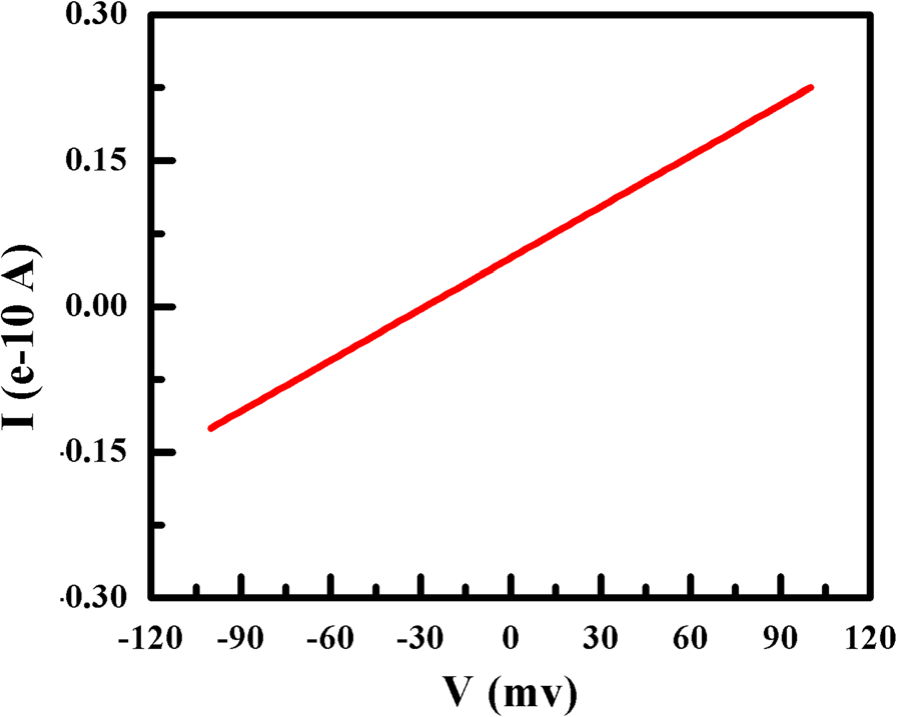
The electrical transport measurement of α-SiNW in Fig. 3. Two-terminal device was used to measure the resistivity of nanowire [25]. The wire was mechanically removed from the substrate by nano-operator equipped on focused ion beam (FIB) (FEI, QUANTA3D 600FIB System). Then, nanowire was weld on the two electrodes by Pt deposited with assisted electron beam. The resistivity of the nanowire was measured by Cascade Semi-automatic probe station HP 4156. Finally, the room temperature resistivity of the nanowire in Fig. 3 is 2.15 × 10^3^ Ω·cm, measured by two-probe method

Figure 10 shows the current (*I*) versus voltage (*V*) fitting curve with a near ohmic behavior. *I–V* measurement shows the room temperature resistivity; the nanowire in Fig. 3 is 2.15 × 10^3^ Ω·cm, measured by two-probe method. Comparing with M. Lieber’s work [29], the resistivity of α-SiNW in this work is ten times higher than the single crystal silicon wires without doping. The significantly higher electrical conductivity of the nanowires is probably due to the size effect.

## Conclusions

In conclusion, α-SiNWs are grown directly on SiO_2_ surface during annealing process in Ar/H_2_ atmosphere via SLS mechanism without any toxic precursor gases. Cu patterns fabrication is the necessary condition for α-SiNW growth. Meanwhile, Cu patterns are used to control the density and the site of α-SiNWs. What is more, the annealing time is adjustable parameters to control the diameter and length of wire α-SiNWs. The room temperature resistivity of the nanowire is 2.15 × 10^3^ Ω·cm. This new growth method makes α-SiNWs candidate for potential applications in the future.
